# The epidemiology and clinical features of the neglected myiasis: A pilot surveillance study from Oman

**DOI:** 10.5339/qmj.2024.15

**Published:** 2024-03-14

**Authors:** Zayid K. Almayahi, Mahfoudha Al Hattali, Zalkha Al Kharusi, Dalal Al Shaqsi, Khairy Anis

**Affiliations:** 1 Disease Surveillance and Control Department, Ministry of Health, South Batinah, Rustaq, Oman Email: almayahi96@hotmail.com; 2 Wadi Mistal Hospital, Primary Health Care Department, Ministry of Health, South Batinah, Wadi Mawel, Oman

**Keywords:** Oman, myiasis, surveillance, neglected disease, zoonotic disease, one health

## Abstract

Background: Myiasis is a neglected disease, characterized by ill-defined diagnostics and management protocols. Published epidemiological and clinical studies of myiasis are still scarce, although several countries, such as Oman, have reported a few cases over the past 30 years. This study explores the epidemiological profile and clinical characteristics of myiasis in South Batinah Governorate (SBG), Oman.

Methodology: A prospective surveillance study was conducted in SBG from 1st November 2018 to 31st October 2019. Clinical and epidemiological data were collected using a pre-designed reporting form for suspected and confirmed cases.

Results: A total of 188 cases were reported, of which 81.4% were male. One-third of cases were 11–20 years old, and a quarter reported exposure inside house. The rate of exposure was 16 per 10000 in Nakhal and Wadi Mawel. About 57% patients reported multiple re-exposure. Almost 60% of patients developed nasal or oral myiasis, 25% developed myiasis in the eye, and 4% in the ear. The most common respiratory symptoms were cough (83%), runny nose (48%), and foreign body sensation (35%). Over 50% of patients with eye exposures had redness, pain, and itchiness. Most patients (89.3%) kept animals at homes. Many cases occurred between December 2018 and April 2019, with almost one-third of exposures occurring at 5 p.m. Re-exposure likelihood for patients residing in Nakhal and Wadi Mawel was high; OR = 2.49 (95% CI 1.14–5.45), with OR = 3.59 (95% CI 1.37–9.39) for 11–20-year-olds and, OR = 3.01 (95% CI 1.07–8.42) for patients ≥ 31 years.

Conclusion: The myiasis exposure rate is high in certain areas and age groups, which is most likely associated with animal-related activities. The disease has a significant impact upon people’s health in SBG. Therefore, urgent active-surveillance and clinical studies are warranted to explore possible preventive measures and treatment options. Adopting one health approach could offer an effective strategy for preventing myiasis in human and animal populations.

## Introduction

Myiasis is defined as the infestation on humans and vertebrate animals by dipterous larvae.^1^ This can occur in different body parts, including but not limited to eyes, nose, ears, lungs, and cutaneous wounds. Over the past three decades, several human cases have been reported in Oman, which have mainly been caused by adult female *Oestrus ovis* (Diptera: Oestridae), commonly known as the sheep bot fly. It can cause severe parasitosis in sheep and goats, and occasionally other species of animals too.^2^ A systematic review published in 2019 showed a high estimated global prevalence of oestrosis (myiasis caused by *Oestrus ovis*) in sheep and goats, reaching up to 48.25% (95% CI 41.82–54.67%).^2^ While *Oestrus ovis* is found worldwide, oestrosis is endemic in hot and dry areas.^3^ Unsurprisingly, the disease has a negative impact on the productivity of sheep, causing severe economic losses in affected regions.^4^

Although high numbers of myiasis of varying severity and symptoms have been reported in humans in many areas of the world, there are few structured research studies of the disease.^1,5–13^ As a result, gaps in the knowledge of the disease’s epidemiology, clinical manifestations, and effective management protocols persist.^1^ Researching the disease is made challenging by some healthcare professionals underestimating the significance of the disease, regarding it to be of minor importance; therefore, the disease is likely to be under-reported. Furthermore, the reliable identification of maggots and fly species is constrained by poor access to entomological services, particularly in developing countries.^1^

Consequently, there is a need to understand in detail all aspects of this disease to arrive at effective prevention and treatment strategies. This study aimed to pilot a surveillance system for the disease, identify the clinical features and potential risk factors for acquiring the infection, and examine the factors associated with multiple episodes of the disease.

## Methodology

### Setting and Design

A prospective surveillance study was conducted by the Disease Surveillance and Control Department (DSC) of Oman Ministry of Health from 1^st^ November 2018 to 31^st^ October 2019 in SBG; this is located in the northern side of Oman, west of Muscat governorate, and north of Dakhilyah governorate. The climate of SBG is subtropical and dry. The governorate is divided into six districts: Rustaq, Barka, Musana, Nakhal, Wadi Al Maawil, and Awabi. Barka and Musana are coastal districts located next to the Sea of Oman, while the other districts have mountainous and plain terrains. The population size of SBG is estimated at 465,550, based on the 2020 census.^14^ There are a total of 21 primary health institutions distributed across the six districts, with the General Directorate of Health Services and the reference secondary hospital, both located in Rustaq district.

### Data Collection

A pre-designed reporting form was developed using the Epi Info software. The form was divided into two sections. Section A collected demographic data, details about exposure, symptoms, and risk factors. Meanwhile, section B recorded the doctors’ examination findings, diagnosis, treatment, referral, and follow-up details. The reporting form was then reviewed by two public health experts with a medical background and face-validated by piloting it for a few weeks before the study for doubtful or confusing items. We approached the nurses in charge at the health facilities to obtain the data on our behalf. Nurses received full orientation about the project, its importance, and the methodology; these nurses, in turn, conducted briefing sessions for healthcare workers in their respective health facilities.

Two technical staff (authors) from DSC maintained regular contact with the primary health centres, providing reminders about the surveillance project and obtaining monthly summaries about cases.

### Surveillance

Myiasis disease has been observed in the mountainous areas of SBG for long time; however, it has never been added to the list of reported diseases and it does not appear to have been a topic of research.^15,16^ People living in areas where myiasis is prevalent were familiar with the disease and they would seek medical consultation for some symptomatic treatment. A suspected case of myiasis was defined as any case with suggestive symptoms of myiasis on eyes, nose, ears, mouth, or skin wounds, with possible exposure to the flies. Cases were confirmed by the observation of larvae on the patient. Doctors were advised to report the case by filling section A of the form, and to refer patients to the secondary hospital for specialist consultation and management, where section B was filled. Notifications of each case were sent to the DSC for monitoring and updating the disease database.

### Statistical Analysis

The Epi Info software (version 7; Centers for Disease Control and Prevention [CDC]; Atlanta, Georgia, USA) was used to organize and tabulate the data. An Excel spreadsheet file was extracted and used in IBM SPSS 23.0 (IBM Corp.; Armonk, New York, USA). Categorical data consisted of frequencies and percentages. Binary logistic regression was used to determine the association between several demographical and risk factors with each individual’s number of exposures to myiasis. Nakhal and Wadi Mawel were combined as one district for analysis purposes, as both are geographically and administratively interrelated. The odds ratios (OR) and 95% confidence intervals (CI) with bivariate and multivariate analyses were calculated. *P*-values of < 0.2 used for the multivariate analyses; however, only *P*-values ≤ 0.05 were considered statistically significant.

## Results

**Figure 1.**
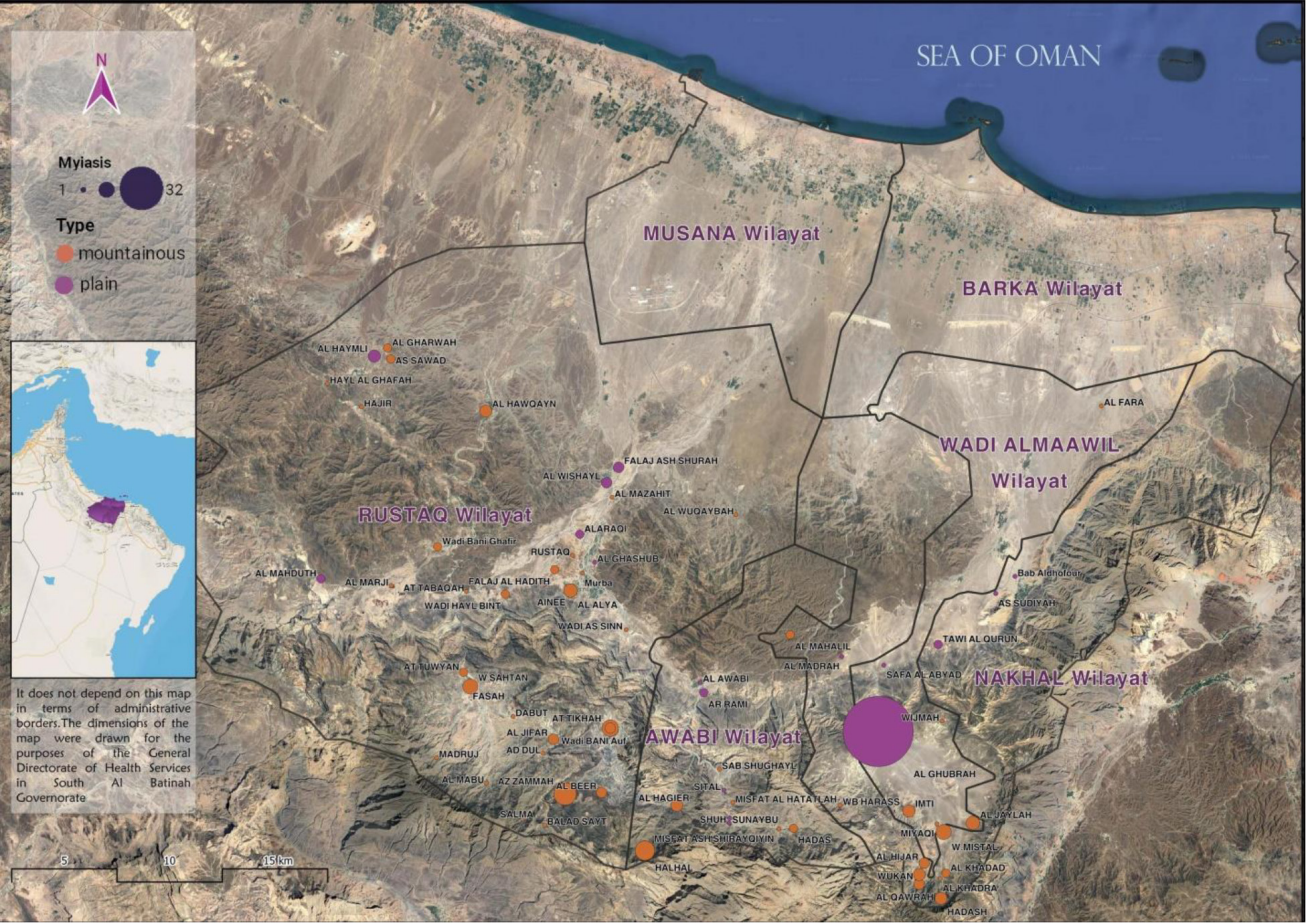
Geographical distribution of the reported cases across SBG.

**Figure 2.**
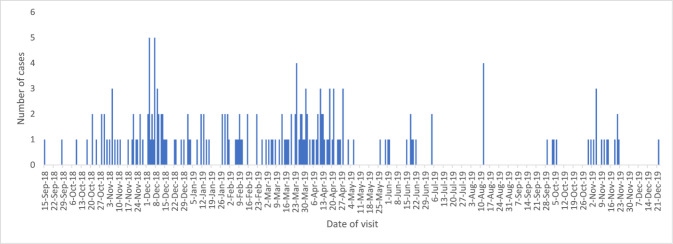
Cases of Myiasis in South Batinah during the study period, by date of notification.

**Figure 3.**
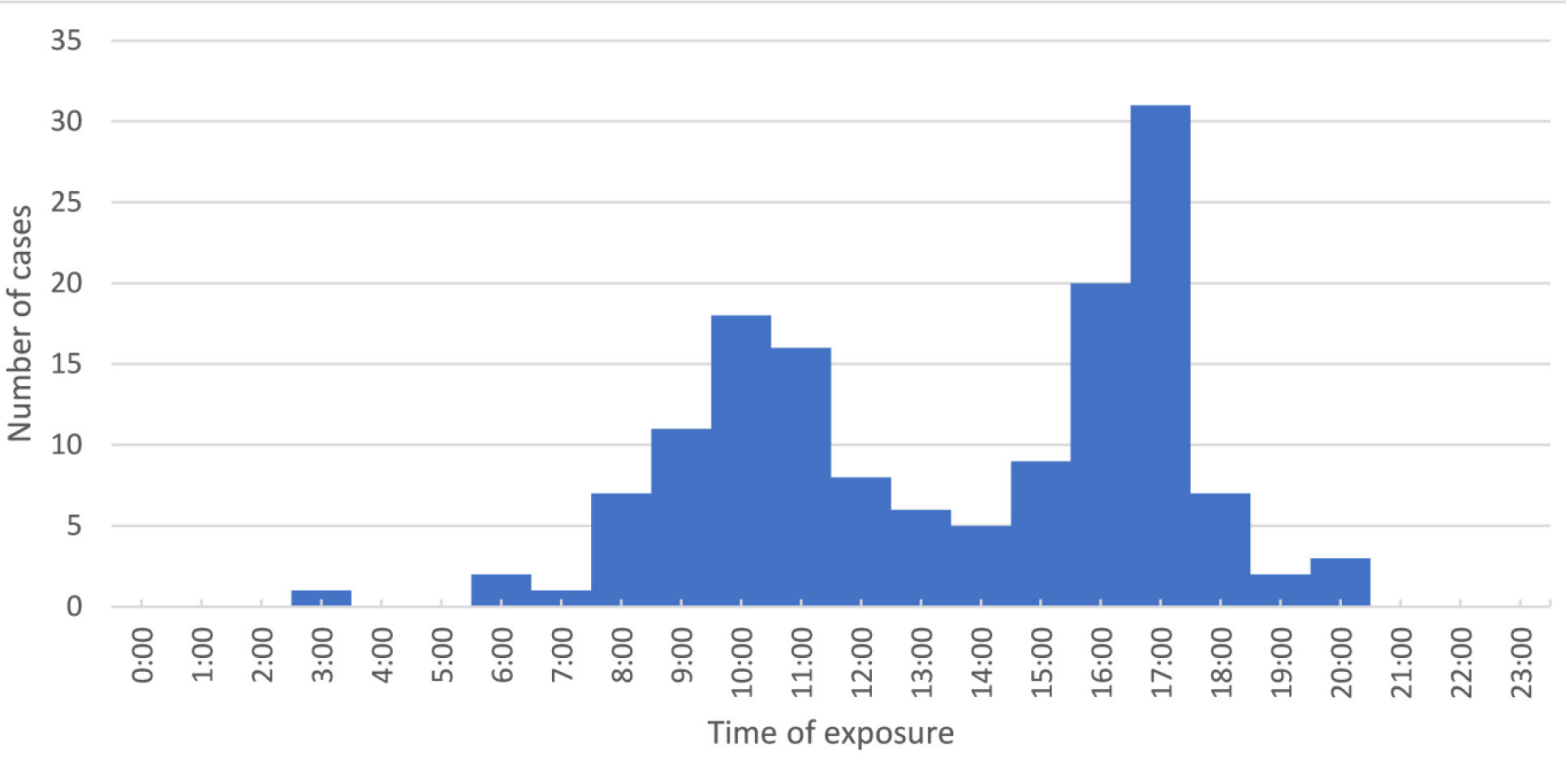
Exposure time to Myiasis.

**Figure 4.**
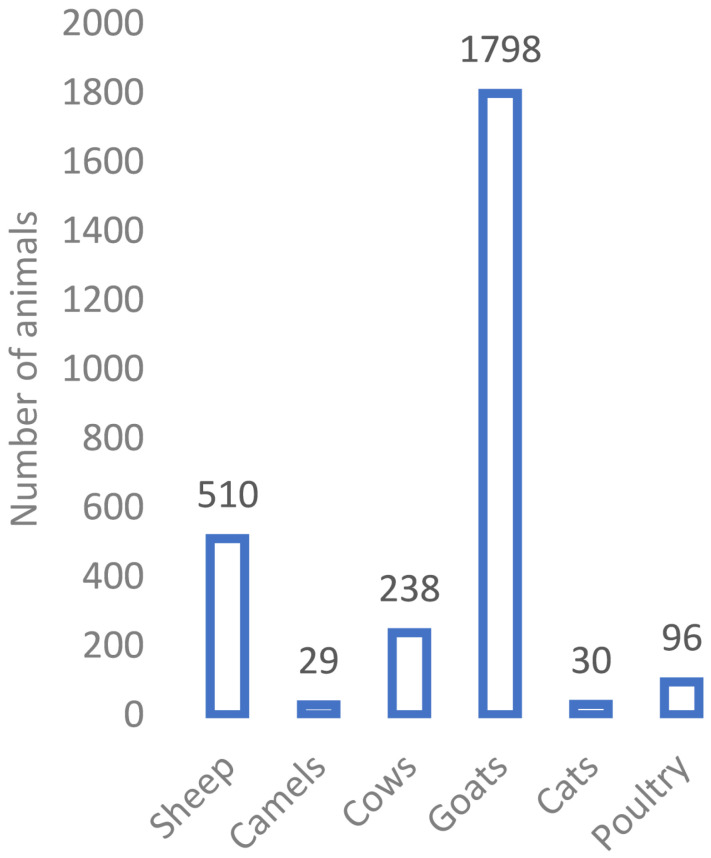
Cumulative total of reported animals, type and number (approximate).

The total number of reported suspected cases was 188, of which 11 were during the piloting phase; 14 were reported after the end of the study. The median age (interquartile range) was 19 years (11–32 years). One-third of cases were in individuals aged between 11 and 20 years, followed by children aged 10 years or less (22.3%), while 81.4% of cases were male. About a quarter of cases had school certificate or undergraduate degree. Most of the cases were from Rustaq (45.2%) followed by Nakhal and Wadi Mawel (41%), where the exposure rate was 16 per 10,000 population. Almost a quarter of patients reported exposure inside the house, while the majority of patients (56.4%) had already reported several episodes of myiasis infection in the past. The median number (interquartile range) of previous exposures was 3 (2–5). Almost 60% of patients developed nasal or oral myiasis, followed by eye (25%) and ear (4%) myiasis; 24 patients reported possible mixed myiasis (Table 1). Figure 1 depicts the geographical distribution of cases based on district and terrain type. The most common respiratory symptoms for mouth and nose exposure were cough (83%), runny nose (48%), and foreign body sensation (35%). The most common symptoms for ear exposure were ear pain (75%), itchiness (54%), and foreign body sensation (42%). Over 50% of patients with eye exposures experienced redness, pain, and itchiness. Most patients (89.3%) owned animals at home. Thirty-two percent of patients were either taking care of animals or had been on farms when they were exposed. Only 16% of cases were referred for specialist consultation. Eye inspection of one case had obvious maggots, while examination with an ophthalmoscope identified maggots in five cases. The median number of maggots seen was six. Most common findings reported for eye exposure were eyelid oedema, chemosis, discharge, redness, and congestion. Most common diagnosis given by ophthalmologists was conjunctivitis and foreign body, while ear, nose, and throat specialists’ common diagnoses were acute laryngopharyngitis, otitis media, allergic rhinitis, and upper respiratory infection (Table 2). Figure 2 shows the distribution of cases throughout the reported period. A high number of cases were reported at the beginning of December through the end of April. Exposures mainly occurred between the hours of 8 a.m. and 12 p.m. and between 3 p.m. and 6 p.m. Almost one-third of exposures took place at 5 p.m. (Figure 3). Goats, sheep, and cows were the most common types of animals owned by patients, (*n* = 1798, *n* = 510, and *n* = 238 respectively) (Figure 4). The odds of experiencing multiple episodes were higher in patients aged between 11 and 20 years (OR = 3.59; 95% CI 1.37–9.39), and 31 years or older (OR = 3.01; 95% CI 1.07–8.42). Patients residing in Nakhal and Wadi Mawel were the most vulnerable to multiple episodes (OR = 2.49; 95% CI 1.14–5.45) (Table 3).

## Discussion

This is the first myiasis surveillance study in Oman and the Arabian Peninsula region among human population that aims to assess the extent of myiasis from both clinical and public health perspectives. The study followed a surveillance approach in 2018/19 throughout the SBG, which is comprised of six districts. The disease has been reported only in the four mountainous districts; no cases have been reported in the two coastal districts. For various reasons, only 30 patients were referred for specialist consultation. Reasons included patient refusal, as well as logistical challenges of accessing the reference specialist medical centre, which was located distantly.

Inhabitants of some of the mountainous areas in Oman had already informed the authorities concerned about the disease several times to help control the bot flies.^15,16^ Patients are familiar with the disease and the causative organism *Oestrus ovis* of the Oestridae family. Indeed, our research has also revealed some of the traditional therapeutic approaches practiced by sufferers, of which some could be seriously harmful, such as applying fuel oil, mercury, insecticides, and cigarettes. These treatments might explain why most of the larvae were not observed in majority of patients. The efficacy of traditional therapies could be an interesting focus of future studies. At present, there is no standard protocol for the treatment worldwide, apart from the symptomatic medicines and manual removal of maggots.^17,18^ Several myiasis cases were reported previously in Oman, indicating the disease’s long existence in the country. The most recent case was bronchial myiasis diagnosed in a 13-year-old boy from a mountainous village in the interior of the country. The child developed severe respiratory distress and anaphylactic shock. Cytological analysis of bronchial aspirate identified *Oestrus ovis* larvae as the causative organism.^10^ A case of opthalmomyiasis was diagnosed in a 21-year-old male student, who acquired the infection during a field trip in a mountainous village in Muscat; 60 *Oestrus ovis* maggots in the first instar larval stage were removed from the affected eye.^9^ Two cases of oral myiasis were also diagnosed in 12- and 21-year-old females, who both had cerebral palsy and incompetent lips; a large number of maggots were found under the surrounding mucoperiosteum.^8^

We understand that since people are familiar with the disease and aware that treatment is limited to some symptomatic medicines, there are possibly dozens of cases that have not been reported to the health facilities, making the estimation of incidence rate extremely difficult. Therefore, future studies should explore the feasibility of implementing active surveillance strategies to identify undetected cases and to acquire reliable data about the incidence and prevalence of myiasis.

Myiasis is typically more prevalent in tropical and subtropical areas, especially those with warm and humid conditions.^19^ It is known that myiasis can affect people of any age; however, most of our cases were male in their second decade of life, followed by patients over 31 years. These findings are consistent with those reported in a systematic review.^17^ Indeed, these two age categories also carried the risk of multiple myiasis infection. The reason why the incidence is lower in 21–30-year-olds could be that many people in that age group are enrolled in undergraduate studies and work outside SBG. Patients living in Nakhal and Wadi Mawel are more likely to get re-exposure, which could be explained by the high population of sheep and goats in these districts and subsequently the abundance of bot fly. However, there could be other reasons including climate conditions being more favorable to bot flies.

A high number of patients reported having animals at home or being exposed to animals and farm activities. A previous study from Libya identified exposure with different animals for all 21 cases diagnosed with external opthalmomyiasis due to *Oestrus ovis*.^20^ The two most common animal species owned by people living in the four districts as shown in our results are goats and sheep.

Although the literature suggests that a low socioeconomic level is an important risk factor for acquiring the infection, our results do not suggest it.^21^ Findings also showed higher exposure rates during winter, which possibly reflect the seasonal and the peak period for fly activity in the SBG. A study on the kinetics of *Oestrus ovis* infection in northern Spain found that adult instars appear in May and persist until November, while there is a diapause beginning in October–November that lasts until February.^22^ Unsurprisingly, patients reported exposure time mainly occurring at 10 a.m. or 5 p.m., when most of outdoor activities occur, including taking care of animals, farms, and other outdoor activities. Most patients in our study acquired myiasis through exposure to the nose, mouth, or eye, while the least had aural myiasis. This agrees with the findings of two otorhinolaryngological myiasis studies from India published in 2009 and 2020.^7,23^ However, another study published in 1993 found that 86% of cases were aural infections, 12% were nasal, and only 2% were ocular.^24^ In general, the reported symptoms in our study, including the systematic symptoms, are in accordance with the literature.^1^

People living in areas with high exposure rates, such as in our study, have had long adaptation to such conditions, and many of them may not be keen to seek medical intervention. However, the severity of the disease can be significant in individuals, especially the elderly, bed ridden, people with disabilities, chronic conditions, immunocompromised, psychiatric patients, and children. Myiasis may be undetected for a long time in any of these patients, leading to critical complications. Recently, tracheostomal myiasis was reported in a 4-year-old child with Guillain–Barre syndrome, vaginal myiasis in a 67-year-old homeless woman with multiple comorbidities, and cutaneous myiasis in a 95-year-old woman with a history of basal cell carcinoma.^25–27^

Unfortunately, myiasis has been regarded to be a neglected health disease across different parts of the world for many years. Moreover, the modelling studies and expert reviews signal a strong tendency toward increased endemicity of some myiasis-causing fly species, making the future even more challenging.^28–30^ Thus, neglected diseases including myiasis should be re-evaluated thoroughly and urgently. Health policies could prioritize this disease under the surveillance system and establish the required monitoring protocols.

While we argue that there is a need for more research to be conducted to understand the different risk factors for myiasis infection in humans, we consider there to be an urgent need to develop a one health approach to address this challenge from a public health perspective. Health departments should be working hand-in-hand with animal, agricultural, and environmental sectors to plan for collaborative surveillance and control efforts. The joint work could result in the development of effective tools, and an extensive surveillance umbrella for humans, animals, and entomological services. In addition, an interdisciplinary approach would facilitate the accurate exploration and identification of the root causes, risk factors, and other related aspects of myiasis.

At the same time, further studies are required to search for the best treatment modalities for humans and animals, while also raising the awareness and educational campaigns about basic personal protective equipment and other possible preventive measures, especially when dealing with animals where bot flies are abundant. Those interventions should be prioritized for populations most vulnerable to exposure to the flies and at risk of medical complications from the disease. Using our study as a springboard, there are several diverse aspects to preventive measures and therapeutic options for human and animal populations that future studies could explore.

## Limitation

This study has several limitations. First, most patients were not referred for specialist evaluation for various reasons. We understand that only 16% of cases were referred, while larvae were detected in few of them. The reason why larvae were not detected in some could have resulted mainly from the traditional treatment people used at home. However, the historical knowledge and awareness of the disease among vulnerable populations, along with the propensity for reinfection, means that there is an extremely high likelihood that the suspected cases are indeed positive. Second, the socioeconomic status of patients was evaluated subjectively; the robustness of future work will be enhanced by evaluating this metric objectively. Third, the passive surveillance mechanism followed in this study leaves many other patients undetected at homes, yet the number of reported cases is relatively high. Future studies should consider the option of active surveillance or awareness and educational campaigns to help diagnose and guide more cases. This will also present an opportunity to assess the sensitivity and effectiveness of the ongoing passive surveillance system of such diseases. Fourth, the seasonal or temporal trend noted in our study could have been biased by the underreporting of cases, especially those undetected at home. This is another limitation that can be minimized by future active surveillance studies.

## Conclusion

The high number of cases reported in this study indicates that myiasis is common and has a significant impact upon people’s health in SBG. The risk of myiasis is highest among people working with animals or on farms, while re-exposure is more likely to occur in particular areas and ages. More research is urgently needed to fill the knowledge gaps present in preventive and clinical areas; these studies should preferably use active surveillance approaches. Meanwhile, the one health approach could provide strategies that prevent myiasis in both human and animal populations.

## Ethics Approval

The study received ethical approval from the Research and Ethical Review & Approve Committee at SBG on 12th August 2018. The research number is 02032018. Patients’ personal data, the medical investigations, and outcomes were collected and analyzed anonymously.

## Conflicts of Interests

The authors have no conflicts of interest to declare.

## Availability of Data

The datasets are not publicly available but are available from the corresponding author upon reasonable request.

## Authors’ Contributions

Conceptualization: ZKA; Data curation: ZKA, MAH, ZAK, DAS; Formal analysis: ZKA; Investigation: ZKA, MAH, ZAK; Methodology: ZKA; Project administration: ZKA; Supervision: ZKA, MAH; Validation: ZKA, ZAK; Writing–original draft: ZKA; Reviewing & editing: all authors. All authors read and approved the final manuscript.

## Acknowledgments

We would like to express our gratitude to the nurses in charge in primary health care facilities in SBG for their time, efforts, and dedication to this project. We greatly appreciate the excellent and pivotal role of our general practitioners, ophthalmologists, and ENT specialists in SBG. We thank Dr. Khalid Nasr Alsaid Abdalhlim from the Ministry of Agriculture, Fisheries and Water Recourses (Rustaq veterinary clinic) for his great insight about the project and the paper. Thanks and appreciation to Ms. Marwa Al Khudhuri from the Planning and Studies department for supporting mapping the cases via GIS. Likewise, many thanks to our supportive colleagues from the Disease Surveillance and Control in SBG for their encouragement, support, and insight.

## Figures and Tables

**Table 1.** Demographical characteristics of cases.

**Variable**	**Total No**	**%**
**Age (years)**		
≤ 10	42	22.3
11–20	62	33
21–30	35	18.6
31–40	18	9.6
41–50	16	8.5
51–60	10	5.3
61–70	3	1.6
> 70	2	1.1
Median + (IQR)	19 (11–32)	
**Gender**		
Male	153	81.4
Female	35	18.6
**Social status**		
Single	134	71.3
Married	49	26.1
Missing	5	2.7
**Education**		
None or early childhood education or school student	125	66.5
Completed secondary school or college degree	49	26.1
Missing	14	7.4
**Place during exposure**		
Inside house	49	26.1
Outside house	136	72.3
Missing	3	1.6
**Frequency of episodes**		
First-time episode	67	35.6
Multiple episodes	106	56.4
Missing	15	8
**Routes of infections**		
Nose/Mouth	111	59
Eye	46	24.5
Ear	7	3.7
Mixed infections(nose/mouth/eye/ear)	24	12.8
**Wilayat**	N (%)	Rate/10000
Rustaq	85 (45.2)	7.2
Awabi	26 (13.8)	13.8
Nakhal & Wadi Mawel	77 (41.0)	16.0

**Table 2.** Distribution of cases based on the main route of exposure and symptoms, risk factors, and referred case characteristics.

**Route of Exposure and Respective and Systematic Symptoms**										
Route of exposure*	N	Symptoms	N	%	Symptoms	N	%	Symptoms	N	%
**Mouth/Nose**	128	Cough	106	83	Sneezing	22	17	Epistaxis	0	0
		Runny nose	62	48	Dyspnea	15	12	Foul smell	0	0
		Foreign body sensation	45	35	Wheeze	9	7			
		Sore throat	44	34	Odynophagia	2	2			
		Itchiness feeling	30	23	Anosmia	0	0			
**Ear**	24	Pain EAR	18	75	Tinnitus	2	9	Bleeding	0	0
		Itchiness	13	54	Discharge	2	9			
		Foreign body sensation	10	42	Foul smell	0	0			
**Eye**	65	Redness	44	68	Foreign body sensation	26	40	Photophobia	2	3
		Eye pain	38	59	Eye Discharge	18	28			
		Eye Itchiness	34	52	Eye Swelling	19	29			
**Body symptoms**	188	Nausea Vomiting	21	11	Body pain	4	2	Constipation	0	0
		Headache	21	11	Dizziness	1	1	Diarrhea	0	0
		Fever	16	9	Body itchiness	2	1	Body rash	0	0
**Other risk factor specifications**					**N (%)**					
Patients were outside house during exposure					136/185, (73.5)					
Patients were in the farms or caring for animals during exposure					54/171, (31.6)					
Patients who had comorbidities					13/188, (6.9)					
Patients having animals in their houses					150/168 (89.3)					
**Referred cases characteristics**					**N (%)**					
Number of cases referred					30/188, (16.0)					
Maggots obvious on eye inspection					1/30, (3.33)					
Maggots obvious on ophthalmoscope					5/30, (16.7)					
Median number of maggots seen					6					
(Eye) Examination findings					Eyelid oedema, chemosis, discharge, redness, congestion,					
(Eye) Major diagnosis					Conjunctivitis, Foreign body					
(Eye) Major treatment offered					(Fusidic acid, ofloxacin, tetracycline, fluorometholone), dexamethasone					
(ENT) Examination findings					Congested oropharynx					
(ENT) Major diagnosis					Laryngopharyngitis, otitis media, allergic rhinitis, Upper respiratory infection,					
(ENT) Major treatment offered					Mouth wash, Antibiotics (ciprofloxacin, cefalexin), fluticasone					

* Route of exposures considered for patients with mixed exposures.

**Table 3.** Patient characteristics, unadjusted and adjusted odds ratios and 95% confidence intervals for the likelihood of multiple episodes of myiasis.

**Variable**	**Total No**	**One-Time episode** ***N* = 67**	**Multiple episodes** ***N* = 106**	**OR (95% CI); *P*-value Bivariate analysis**	**OR (95% CI); *P*-value Multivariable analysis**
**Median (IQR) previous episodes**		–	3 (2–5)	–	–
		No. (%)	No. (%)		
**Age (years)**					
≤ 10 (Reference group)	39 (23)	22 (56.4)	17 (43.6)	1	1
11–20	55 (32)	14 (25)	42 (75)	**3.88 (1.62**–**9.32); 0.002**	**3.59 (1.37**–**9.39); 0.009**
21–30	33 (19)	17 (51.5)	16 (48.5)	1.22 (0.48–3.09); 0.678	0.90 (0.32–2.54); 0.835
≥ 31	45 (26)	14 (31.1)	31 (68.9)	**2.87 (1.17**–**7.00); 0.021**	**3.01 (1.07**–**8.42); 0.036**
**Gender**					
Male	141 (81.5)	53 (37.6)	88 (62.4)	1	–
Female	32 (18.5)	14 (43.8)	18 (56.3)	0.77 (0.36–1.68); 0.519	–
**School**					
None or early childhood education or school student	116 (71.6)	44 (37.9)	72 (62.1)	1	–
Completed secondary school or college degree	46 (28.4)	16 (34.8)	30 (65.2)	1.15 (0.56–2.34); 0.708	–
**Wilayat**					
Rustaq	79 (45.7)	36 (45.6)	43 (54.4)	1	1
Awabi	25 (14.5)	9 (36.0)	16 (64.0)	1.49 (0.59–3.77); 0.401	1.41 (0.48–4.10); 0.533
Nakhal & Wadi Mawel	68 (39.9)	22 (31.9)	47 (68.1)	1.79 (0.91–3.50); 0.090	**2.49 (1.14**–**5.45); 0.022**
**Mountainous/Plain**					
Plain	22 (12.7)	8 (36.4)	14 (63.6)	1	–
Mountainous	150 (87)	59 (39.1)	92 (60.9)	0.89 (0.35–2.25); 0.808	–
**Family members**					
1–5	18 (16.1)	9 (50)	9 (50)	1	–
6–10	57 (50.9)	23 (40.4)	34 (59.6)	1.48 (0.51–4.29); 0.472	–
≥ 11	37 (33)	14 (37.8)	23 (62.2)	1.64 (0.53–5.13); 0.393	–
**Animals in the house**					
No	16 (9.9)	8 (50.0)	8 (50.0)	1	–
Yes	145 (90.1)	54 (37.2)	91 (62.8)	1.69 (0.60–4.75); 0.324	–
**Slaughtering Animal**					
No	156 (96.3)	60 (38.5)	96 (61.5)	1	–
Yes	6 (3.7)	2 (3.2)	4 (4)	1.25 (0.22–7.03); 0.800	–
**Dealing with Animals or Farms**					
No	109 (67.3)	47 (43.1)	62 (56.9)	1	1
Yes	53 (32.7)	15 (28.3)	38 (71.7)	1.92 (0.95–3.90); 0.071	2.05 (0.89–4.72); 0.091
**Location while exposure**					
In the house	49 (28.3)	19 (28.4)	30 (28.3)	1	–
Outside house	124 (71.7)	48 (38.7)	76 (61.3)	1.00 (0.51–1.98); 0.994	–
**Socioeconomic status**					
Low	18 (13.7)	8 (44.4)	10 (55.6)	1	–
Medium/ high	113 (86.3)	44 (38.9)	69 (61.1)	1.25 (0.46–3.42); 0.658	–

Note: The bold font is statistically significant.
